# A National Analysis of Microscopic Positive Margins in Oropharyngeal Cancer Patients Undergoing Transoral Robotic Surgery

**DOI:** 10.1002/ohn.70100

**Published:** 2026-01-26

**Authors:** Aaron Tucker, Craig A. Bollig

**Affiliations:** ^1^ Department of Otolaryngology–Head and Neck Surgery Rutgers Robert Wood Johnson Medical School New Brunswick New Jersey USA

**Keywords:** adjuvant chemoradiotherapy, HPV, oropharyngeal cancer, overall survival, positive margin, transoral robotic surgery

## Abstract

**Objective:**

The objective was to identify clinical variables associated with microscopic positive margins (PMs) during transoral robotic surgery (TORS) for oropharyngeal cancer (OPC) resection, and to explore the association of the receipt of adjuvant treatment with overall survival (OS) in this population.

**Study Design:**

Retrospective cohort analysis.

**Setting:**

2019 Patient User File of the National Cancer Database.

**Methods:**

Patients >18 years of age with OPC were stratified based on margin status. Multivariable logistic regression was used to identify clinical variables associated with PM. Survival analyses were performed using multivariable Cox proportional hazards models. Adjusted odds ratios (aORs) and hazard ratios (aHRs) with associated 95% confidence intervals (CIs) were generated.

**Results:**

In total, 4294 patients met the criteria. The PM rate was 16.6%. Human papillomavirus (HPV)‐negative squamous cell carcinoma (SCCa), salivary gland carcinoma, clinical T category, base of tongue primary site, and treatment at low‐volume, nonacademic institutions were independently associated with PM. PMs were associated with increased mortality (aHR 1.67, 95% CI: 1.40‐1.99). In patients with PM, but without extranodal extension (ENE), adjuvant radiation therapy (aRT) (aHR 0.29, 95% CI: 0.19‐0.45) and adjuvant chemoradiotherapy (aCRT) (aHR 0.31, 95% CI: 0.21‐0.45) were associated with an improvement in OS versus surgery alone; however, OS between aRT and aCRT was similar for both HPV‐positive and HPV‐negative SCCa.

**Conclusion:**

Histologic type, clinical T category, tumor subsite, and treatment at low‐volume, nonacademic institutions were independently associated with TORS PM. aCRT did not confer a survival benefit over aRT in the overall cohort, or in subgroups of HPV‐associated or HPV‐negative SCCa patients with PM without ENE.

Since its Food and Drug Administration (FDA) approval in 2009, transoral robotic surgery (TORS) has become increasingly utilized for the treatment of oropharyngeal cancer (OPC). This minimally invasive approach improves visualization and surgical access to the oropharynx.^1^ Studies over the past decade have highlighted the benefits of TORS over non‐robotic surgery, including lower positive margins (PMs) rates^2^, ^3^, ^4^ and improved overall survival (OS).^4^, ^5^, ^6^, ^7^

In oncologic surgery, the goal is to completely resect the tumor with a cuff of normal, healthy tissue on the margin. There is abundant literature across head and neck surgical oncology literature that PMs are associated with poor prognosis, due to increased recurrence rates and decreased survival.^8^, ^9^, ^10^, ^11^, ^12^, ^13^, ^14^, ^15^, ^16^, ^17^, ^18^ Within the oropharynx, there are anatomic constraints that limit obtaining the 5 mm margins that were conventionally recommended by the National Comprehensive Cancer Network (NCCN) to be considered “clear.”^19^ However, patients with close margins may not have worse oncologic outcomes following TORS, particularly in human papillomavirus (HPV)‐positive patients.^20^, ^21^ Several clinical variables have been shown to be associated with PMs during TORS, including base of tongue (BOT) subsite and tumor T category; however, these remain incompletely analyzed on a national level.^22^, ^23^

PMs are typically an indication for adjuvant treatment, generally adjuvant chemoradiotherapy (aCRT). This recommendation is based on the results of two randomized controlled trials^24^, ^25^ and a meta‐analysis^26^ that showed improved survival with the addition of chemotherapy to adjuvant radiation therapy (aRT) alone in patients with PM or extranodal extension (ENE). However, given the favorable prognosis of HPV‐positive OPC and the significant increase in treatment‐associated morbidity with chemotherapy, some have questioned the utility of treatment intensification with chemotherapy.^27^ The objective of this study was to perform a national analysis to identify clinical variables associated with PM during TORS for OPC resection, and to explore the association of adjuvant treatment administration with OS in this population.

## Methods

After the study was determined to be exempt from institutional review board review by Rutgers Robert Wood Johnson University Hospital in New Brunswick, data were obtained from the NCDB. The NCDB is a national hospital‐based registry maintained as a joint project of the American Cancer Society and the Commission on Cancer of the American College of Surgeons. The NCDB currently captures >70% of newly diagnosed cancers from >1500 facilities in the United States. There are established criteria to certify the quality of the submitted data, as well as an application process to obtain the de‐identified data. After distribution of the data, the Commission on Cancer of the American College of Surgeons and the American Cancer Society are not responsible for the statistical analysis or the conclusions presented by the investigators.

We queried the NCDB for all patients ≥18 years old who were diagnosed with an OPC between 2010 and 2017. These patients were identified by morphologic (histologic) and topographic codes from the International Classification of Disease for Oncology, 3rd edition. The morphologic codes included in the analyses were the following: squamous cell carcinoma (SCCa) including variants (8070‐8076, 8083) and minor salivary gland carcinomas, which were composed of the following: Mucoepidermoid Carcinoma (8430), Adenoid Cystic Carcinoma (8200), Polymorphous Low‐Grade Adenocarcinoma [PLGA] (8525), Adenocarcinoma NOS (8140), and other rare types (8550, 8562, 8310, 8147, 8440, 8480, 8290, 8500, 8980, and 8940). The topographical codes that were used included: BOT (C01.9, C02.4), soft palate (C05.1, C05.2), tonsil/lateral pharyngeal wall (C09.0, C09.1, C09.8, C09.9, and C10.2), and other (posterior pharyngeal wall [C10.3], vallecula [C10.0], and overlapping lesion/not otherwise specified [C10.8, C10.9]). The robotic surgical approach code was used to identify the subset of patients that underwent TORS. Patients with distant metastatic disease (M1) or grossly PMs were excluded. Terminating a procedure with grossly PMs in this patient population rather than shifting to an adjunctive open approach is a far deviation from the standard of care, and is more representative of an endoscopic biopsy or debulking rather than an actual attempt at a definitive resection; therefore, these patients were excluded. Patients with missing data were excluded. Because treating facility type is suppressed for patients <40 in the NCDB, these patients were likewise excluded from the analysis.

### Patient Variables and Statistical Analysis

Baseline patient characteristics included a comparison of age, sex, race, insurance status, facility type, tumor subsite, clinical T and N category, histologic type (HPV‐positive SCCa, HPV‐negative SCCa, unknown HPV SCCa, and salivary gland carcinomas), and the presence of ENE. Patients testing positive for high‐risk HPV types 16 or 18 were classified as HPV‐positive. Patients were considered HPV‐negative if they received an HPV test but were negative for high‐risk HPV types 16 and 18. Patients who had not undergone HPV testing were grouped as unknown HPV SCCa. The NCDB includes a unique facility ID code that allows the calculation of facility treatment volume. Facility volume was calculated as the number of patients with an OPC who underwent definitive treatment with TORS at each facility annually. High‐volume facilities were defined as those with the top 5% of annual treatment volume, while the remainder of the facilities with volumes below the top 5% were considered low‐volume facilities.

Patients were stratified by surgical margin status (positive vs negative). Variables among groups were then compared using the chi‐squared test or Fisher's exact test for categorical variables and two‐sided *t* test or non‐parametric equivalent for continuous variables, depending on the normality of distribution. Multivariable logistic regression models were created to identify clinical variables associated with positive surgical margins. Variables approaching statistical significance (*P* < .10) on univariable testing were then included in the initial multivariable logistic regression model. A backward elimination procedure was used to obtain a model containing only predictor variables whose coefficients were significant at the 0.05 level. Adjusted odds ratios (aORs) and associated 95% confidence intervals (CIs) were calculated for each model.

OS was estimated using the Kaplan‐Meier method. OS was defined as the duration of time from the initial diagnosis to the date of last contact or death. Because ENE is also a high‐risk feature and a traditional indication for aCRT, OS in the ENE‐negative population was compared between margin subgroups and adjuvant treatment subgroups using the log‐rank test. Low numbers of ENE‐positive patients prevented analyses of both ENE subgroups. For a less biased estimate of survival differences, a multivariable Cox proportional hazards model was constructed, adjusting for age, comorbidity score, tumor T and N category, and histologic type. These variables were selected a priori. Adjusted hazard ratios (aHRs) and the associated 95% CI were created for these models. To test the proportional hazards assumption, log‐minus‐log plots were used. For all analyses, the threshold for statistical significance was set at *P* < .05. SPSS v29 software was used for data analysis (SPSS Inc, an IBM Company).

## Results

There were 4294 patients with OPC who underwent TORS and met the inclusion criteria. The median duration of follow‐up was 44.0 months (interquartile range: 29.4‐64.4). Baseline patient characteristics stratified by margin status are detailed in Table 1. Overall, there were 713 (16.6%) patients with PM after TORS. HPV‐positive SCCa were associated with the lowest PM rate (13.6%) in comparison to HPV‐negative SCCa (18.0%), HPV‐unknown SCCa (19.9%), and salivary gland malignancies (24.6%, *P* < .001). The PM rate also increased with clinical T category (T1 = 13.0%, T2 = 18.0%, T3 = 26.7%, T4a = 38.3%, *P* < .001). There were significant differences in the prevalence of PM based on tumor subsite: BOT tumors (18.8%), other oropharyngeal subsites (21.6%), tonsil/lateral pharyngeal wall (14.7%), and soft palate (11.3%, *P* < .001). Additionally, rates of PM differed by facility characteristics: low‐volume (20.1%) versus high‐volume (11.4%, *P* < .001) centers and nonacademic (23.9%) versus academic (15.1%, *P* < .001) facilities. With respect to ENE status, PM rate did not significantly differ between patients with ENE‐positive (20.9%) and ENE‐negative (16.4%, *P* = .149) tumors.

**Table 1:** Baseline Patient Characteristics by Margin Status

	No. (%)		
	Positive	Negative	
Characteristic	n = 713	n = 3581	*P* value
*Age*			.006
Mean (SD)	61.1 (9.3)	60.1 (9.3)	
*Sex*			.909
Male	593 (16.6%)	2972 (83.4%)	
Female	120 (16.5%)	609 (83.5%)	
*Race*			.004
White	635 (16.2%)	3295 (83.8%)	
African American	60 (24.2%)	188 (75.8%)	
Other	18 (15.5%)	98 (84.5%)	
*Insurance status*			<.001
Private	380 (15.0%)	2159 (85.0%)	
Medicaid	57 (23.8%)	183 (76.3%)	
Medicare	247 (18.7%)	1071 (81.3%)	
Uninsured	9 (15.5%)	49 (84.5%)	
Other	20 (14.4%)	119 (85.6%)	
*CDCC*			.001
0	519 (15.5%)	2832 (84.5%)	
1	143 (19.7%)	582 (80.3%)	
2	35 (22.9%)	118 (77.1%)	
3	16 (24.6%)	49 (75.4%)	
*Facility*			<.001
Academic	538 (15.1%)	3025 (84.9%)	
Nonacademic	175 (23.9%)	556 (76.1%)	
*Facility volume*			<.001
High (≥95 percentile)	196 (11.4%)	1520 (88.6%)	
Low	517 (20.1%)	2061 (79.9%)	
*Tumor subsite*			<.001
Tonsil/lateral pharyngeal wall	346 (14.7%)	2006 (85.3%)	
Base of tongue	306 (18.8%)	1320 (81.2%)	
Soft palate	8 (11.3%)	63 (88.7%)	
Other^a^	53 (21.6%)	192 (78.4%)	
*Clinical T category*			<.001
T1	257 (13.0%)	1714 (87.0%)	
T2	357 (18.0%)	1630 (82.0%)	
T3	68 (26.7%)	187 (73.3%)	
T4a	31 (38.3%)	50 (61.7%)	
*Clinical N category*			.368
N0: cN0	214 (16.7%)	1068 (83.3%)	
N+: cN1, cN2, cN2A, c2NB, cN2C, cN3	490 (16.6%)	2469 (83.4%)	
*Histologic type*			<.001
HPV‐positive SCCa	265 (13.6%)	1686 (86.4%)	
HPV‐negative SCCa	231 (18.0%)	1051 (82.0%)	
Unknown HPV SCCa	187 (19.9%)	752 (80.1%)	
Salivary gland carcinomas	30 (24.6%)	92 (75.4%)	
*Extranodal extension*			.149
Negative or unknown	682 (16.4%)	3464 (83.6%)	
Positive	31 (20.9%)	117 (79.1%)	

Abbreviations: CDCC, Charlson‐Deyo Comorbidity Class; HPV, human papillomavirus; SCCa, squamous cell carcinoma; SD, standard deviation.

^a^ Other oropharynx includes posterior pharyngeal wall, vallecula, and overlapping lesion/not otherwise specified lesions.

**Figure 1:**
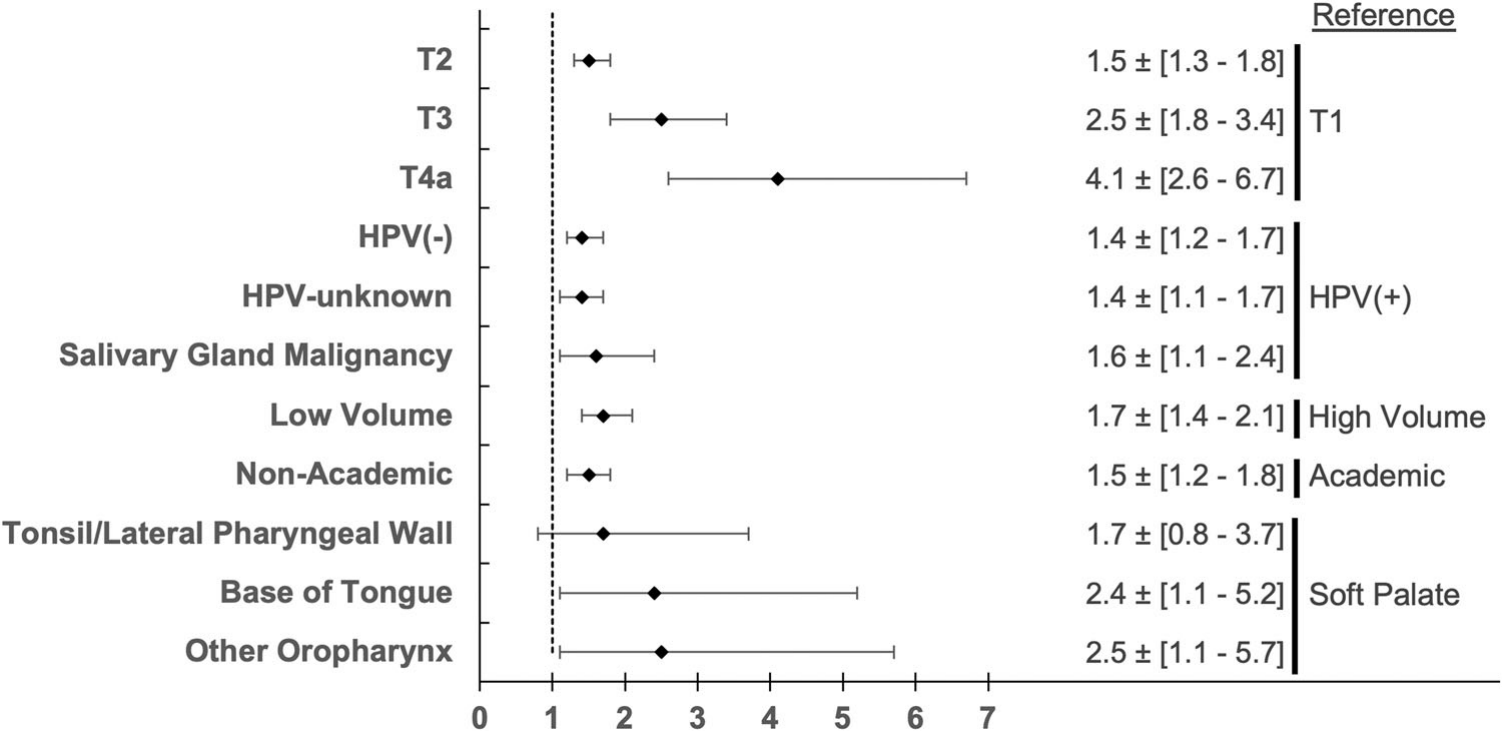
Clinicopathologic and facility predictors of positive margins on multivariable regression analysis. Abbreviation: HPV, human papillomavirus.

Figure 1 details the results of the multivariable analyses aimed at identifying clinical variables independently associated with PM, including facility volume, facility academic status, tumor subsite, tumor category, and histologic type. Low‐volume facilities (aOR 1.7, 95% CI: 1.4‐2.1) and nonacademic status (aOR 1.5, 95% CI: 1.2‐1.8) were associated with PM compared to high‐volume institutions and academic facilities, respectively. Clinical T category displayed a direct relationship with the odds of PM (Reference [ref.] T1; T2: aOR 1.5, 95% CI: 1.3‐1.8; T3: aOR 2.5, 95% CI: 1.8‐3.4; and T4a: aOR 4.1, 95% CI: 2.6‐6.7). Compared to HPV‐positive SCCa, all other histologic types were associated with increased odds of PM (HPV‐negative SCCa, aOR 1.4, 95% CI: 1.2‐1.7; HPV‐unknown SCCa, aOR 1.4, 95% CI: 1.1‐1.7; and salivary gland carcinomas, aOR 1.6, 95% CI: 1.1‐2.5). Finally, relative to soft palate malignancies, BOT tumors (aOR 2.4, 95% CI: 1.1‐5.2) and other oropharyngeal subsites (aOR 2.5, 95% CI: 1.1‐5.7) were associated with increased odds of PM, while tonsil/lateral pharyngeal wall tumors were similar (aOR 1.7, 95% CI: 0.8‐3.7).

**Figure 2:**
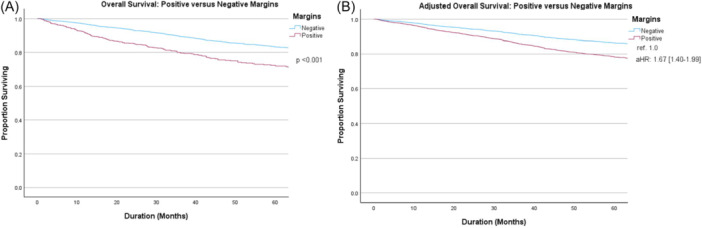
Overall survival of positive versus negative margins on (A) unadjusted and (B) adjusted analysis accounting for age, Charlson‐Deyo Comorbidity Class (CDCC), clinical T and N category, and histologic type. aHR, adjusted hazard ratio; ref., reference.

To better quantify the long‐term prognosis of OPC patients with PM following TORS, we performed survival analyses for histologic and adjuvant treatment subgroups without ENE. Five‐year OS for the entire cohort was 81.5% (95% CI: 80.1%‐82.9%) and significantly differed between those with versus without PM (71.8%, 95% CI: 67.9%‐75.7% vs 83.4%, 95% CI: 81.8%‐85.0%, respectively, *P* < .001) (Figure 2A). Similar findings were seen on adjusted survival analysis (aHR 1.67, 95% CI: 1.40‐1.99, ref. PMs) that accounted for age, Charlson‐Deyo Comorbidity Class (CDCC), clinical T and N category, and histologic type (Figure 2B).

**Figure 3:**
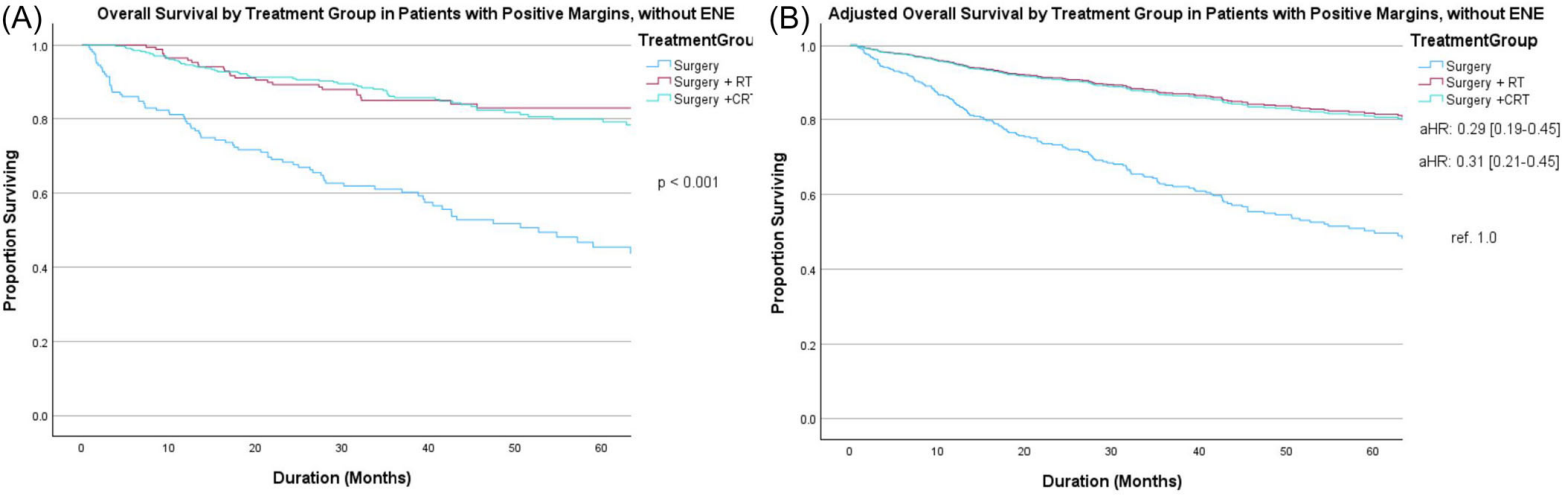
Overall survival in patients with positive margin (PM) without extranodal extension (ENE) by treatment group on (A) unadjusted and (B) adjusted analysis accounting for age, Charlson‐Deyo Comorbidity Class (CDCC), clinical T and N category, and histology. versus chemoradiotherapy (CRT) (reference [ref.] 1.0), radiation therapy (RT) (adjusted hazard ratio [aHR] 0.93, 95% CI: 0.60‐1.45).

aRT (aHR 0.29, 95% CI: 0.19‐0.45) and aCRT (aHR 0.31, 95% CI: 0.21‐0.45) were associated with improved OS in patients with PM without ENE compared to TORS alone (Figures 3A [unadjusted] and B [adjusted]); however, OS did not significantly differ between those treated with aRT (aHR 0.93, 95% CI: 0.60‐1.45) versus aCRT (ref. 1.0).

**Figure 4:**
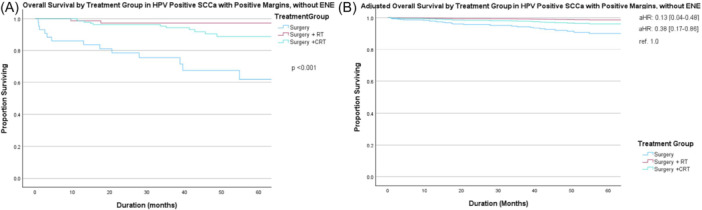
Overall survival by treatment group in human papillomavirus (HPV)‐positive squamous cell carcinoma (SCCa) with positive margin (PM) without extranodal extension (ENE) on (A) unadjusted and (B) adjusted analysis accounting for age, Charlson‐Deyo Comorbidity Class (CDCC), and clinical T and N category. Abbreviations: aHR, adjusted hazard ratio; CRT, chemoradiotherapy; ref., reference; RT, radiotherapy.

**Figure 5:**
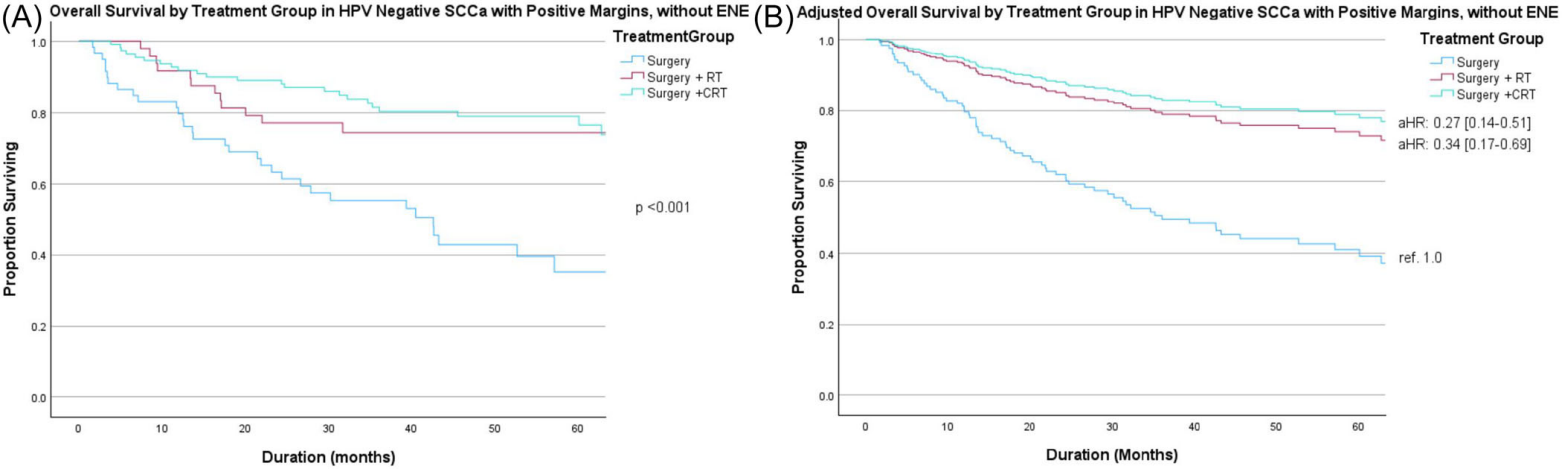
Overall survival by treatment group in human papillomavirus (HPV)‐negative squamous cell carcinoma (SCCa) with positive margin (PM) without extranodal extension (ENE) on (A) unadjusted and (B) adjusted analysis accounting for age, Charlson‐Deyo Comorbidity Class (CDCC), and clinical T and N category. Abbreviations: aHR, adjusted hazard ratio; CRT, chemoradiotherapy; ref., reference; RT, radiotherapy.

Figures 4 and 5 display unadjusted (A) and adjusted (B) survival curves for patients with HPV‐positive and HPV‐negative SCCa and PM without ENE, respectively. aRT and aCRT were both associated with improved OS compared to surgery alone (ref. 1.0) in both HPV‐positive SCCa (aRT, aHR 0.13, 95% CI: 0.04‐0.48; aCRT, aHR 0.38, 95% CI: 0.17‐0.86) and HPV‐negative SCCa (aRT, aHR 0.34, 95% CI: 0.17‐0.69; aCRT, aHR 0.27, 95% CI: 0.14‐0.51), respectively. There was no significant difference in OS between those receiving aRT versus aCRT for either histologic type. The limited number of salivary gland carcinomas with PM was insufficient to allow subgroup analyses on the association of adjuvant treatment in this population.

## Discussion

In this NCDB study of >4000 patients undergoing TORS for OPC resection, we found the national prevalence of PM to be 16.6%. We performed a thorough analysis of clinical variables that are associated with PM. Histologic type, clinical T category, primary site, and treatment at low‐volume, nonacademic institutions were all independently associated with PM. Additionally, we investigated the effect of adjuvant treatment in PM without ENE and found that while adjuvant treatment was associated with improved OS, there was no observed benefit of aCRT compared to aRT alone in any histologic subgroup.

The importance of complete tumor excision with a margin of normal tissue has been well‐validated in head and neck surgical oncology to reduce the risk of recurrence, although the extent of the margin remains a matter of debate.^10^, ^14^, ^16^, ^17^, ^28^ Conventionally, a >5 mm margin was needed be considered “clear,” however, this is not typically feasible within the oropharynx as detailed by Hinni et al.^20^ They noted a mean thickness of the lateral pharyngeal wall of 2.4 mm and a mean deep margin of <2 mm in a prospective series of 128 tonsil cancers resected transorally.^20^ Even with these close margins, a 99% local control rate was achieved.^20^ The current NCCN guidelines mention that margins as close as 1.5 to 2 mm may be acceptable in transorally resected OPC.^19^ Within the phase 2 ECOG 3311 trial, close margins of <3 mm were considered an intermediate risk factor and an indication for aRT.^29^ In a multicenter retrospective study with an even more relaxed definition of close margins (<1 mm or requiring re‐excision), Holcomb et al reported a 100% local control rate in the subgroup of patients with HPV‐positive SCCa undergoing TORS.^21^ As higher‐quality data from prospective TORS trials become available, more insight into the oncologic safety of closer margins will be gained.

In this national data set, the prevalence of PM was 16.6%. This is considerably higher than what is reported in most institutional retrospective reviews. A systematic review and meta‐analysis by Gorphe and Simon identified 3619 patients with OPC undergoing transoral surgery and calculated a PM rate of 7.8%.^30^ Their review identified primarily retrospective studies from high‐volume, academic centers, reflecting a strong publication bias that is present in the current literature. Our results also indicated a significantly lower rate of PM at the top 5% highest volume centers, as well as academic facilities. In an earlier review of the NCDB of the first 4 years following FDA approval of TORS for OPC, Hanna et al found a PM rate of 16.9% nationally and clinically relevant differences between high and low‐volume institutions.^23^ Similarly, Tucker and Bollig reported stepwise decreases in the PM rate between low, intermediate, and high volume facilities, noting that between TORS facility volume and academic status, facility volume was likely the driver of PM rates.^31^ The systematic review by Gorphe and Simon identified several other variables associated with increased risk of PM, including tumor T category (T4 vs T1‐3), the non‐utilization of intraoperative frozen section, and TORS or conventional resection versus transoral laser microsurgery (TLM).^30^ While TORS is currently FDA approved for early‐stage tumors (T1 and T2), select high‐volume institutions have reported favorable results after transoral resection in select patients with larger oropharyngeal malignancies.^32^, ^33^ Increasing tumor size would be expected to be associated with greater rates of PM, and this relationship was seen in our patient cohort as well. OPC subsites differ in their transoral surgical accessibility, with the most direct access to the soft palate, followed by the tonsil and BOT.^22^, ^34^ Accordingly, we saw increasing rates of PM in this pattern. In their early NCDB review of 2010 to 2014, Hanna et al found a trend of differences in PM rates based on HPV status that did not reach statistical significance. They did not include salivary gland carcinomas in their analyses.^23^ With our expanded patient cohort and inclusion of salivary gland carcinomas, there were noteworthy differences in the prevalence of PM based on histologic type. HPV+ SCCa was associated with the lowest rate of PMs in this analysis, followed by HPV− SCCa and salivary gland carcinomas.

PMs are considered to be an indication for adjuvant treatment according to the NCCN guidelines.^19^ This is generally chemoradiotherapy (CRT) based on the results of two randomized controlled trials^24^, ^25^ and a meta‐analysis^26^; however, given the favorable prognosis of HPV‐positive OPC and the significant increase in treatment‐associated morbidity with chemotherapy, some have questioned the utility of treatment intensification with chemotherapy.^27^ In a 2016 single‐center review of 195 patients with p16+ OPC undergoing resection with TLM, Skillington et al demonstrated that aCRT was not associated with improved survival versus aRT alone (5‐year OS 82% vs 84%, respectively).^35^ Similarly, aCRT was not associated with differences in disease‐free survival (79% for both groups) after controlling for age, comorbidity, smoking, and pathologic staging. Their sensitivity analyses determined that in order for aCRT to have a positive effect on OS in HPV+ SCCa, an unmeasured confounding variable would need to be present with an effect size greater than pathologic staging or comorbidity scores. In this national analysis, the administration of adjuvant treatment was associated with improved OS in the overall cohort with PM without ENE, as well as subgroup analyses of HPV+ SCCa and HPV− SCCa with PM without ENE. However, there was no improvement in OS seen in patients with PM without ENE treated with aCRT versus aRT alone in the overall cohort or in subgroup analyses. These results underscore the importance of the need for future randomized controlled trials to address the need for chemotherapy in patients with PM, particularly those with HPV+ disease.

There are several noteworthy limitations with these analyses. The NCDB provides information on the majority of cancers treated in the United States from all types of facilities, both low and high volume; however, it lacks some important clinical information. Survival analyses are limited to OS because the cause of death and information on recurrence are not reported. Inherent to database studies, there is the potential for errors in data entry, missing data, and selection bias. The NCDB also does not provide details of margin distance, which would be beneficial to allow analyses of individuals with close surgical margins. Finally, adjuvant treatment decisions may also be affected by previous treatment, personal choice, or other factors not contained within the NCDB to allow for analysis.

## Conclusion

This national analysis demonstrates that histologic type, clinical T category, tumor subsite, and treatment at low‐volume, nonacademic institutions were all independently associated with PM during TORS. aCRT is not associated with improved OS in patients with TORS PM without ENE compared to radiotherapy alone in OPC overall, HPV‐positive SCCa or HPV‐negative SCCa. Future prospective trials are needed to further define the role of chemotherapy in OPC patients with PM without ENE following TORS.

## Author Contributions

**Aaron Tucker**: design, analysis, interpretation, drafting, revision, presentation of research; **Craig A. Bollig**, design, acquisition, analysis, interpretation, drafting, revision.

## Disclosures

### Competing interests

The authors declare no conflicts of interest.

### Funding source

None.
